# Laboratory Diagnostic Tools for Syphilis: Current Status and Future Prospects

**DOI:** 10.3389/fcimb.2020.574806

**Published:** 2021-02-08

**Authors:** Yuting Luo, Yafeng Xie, Yongjian Xiao

**Affiliations:** Department of Clinical Laboratory, The Second Affiliated Hospital of University of South China, Hengyang, China

**Keywords:** syphilis, laboratory diagnosis, immunohistochemistry, seroassay, nucleic acid amplification technique, polymerase chain reaction

## Abstract

With the increasing number of patients infected with syphilis in the past 20 years, early diagnosis and early treatment are essential to decline syphilis prevalence. Owing to its diverse manifestations, which may occur in other infections, the disease often makes clinicians confused. Therefore, a sensitive method for detecting *T. pallidum* is fundamental for the prompt diagnosis of syphilis. Morphological observation, immunohistochemical assay, rabbit infectivity test, serologic tests, and nucleic acid amplification assays have been applied to the diagnosis of syphilis. Morphological observation, including dark-field microscopy, silver-staining, and direct fluorescent antibody staining for *T. pallidum*, can be used as a direct detection method for chancre specimens in primary syphilis. Immunohistochemistry is a highly sensitive and specific assay, especially in the lesion biopsies from secondary syphilis. Rabbit infectivity test is considered as a sensitive and reliable method for detecting *T. pallidum* in clinical samples and used as a historical standard for the diagnosis of syphilis. Serologic tests for syphilis are widely adopted using non-treponemal or treponemal tests by either the traditional or reverse algorithm and remain the gold standard in the diagnosis of syphilis patients. In addition, nucleic acid amplification assay is capable of detecting *T. pallidum* DNA in the samples from patients with syphilis. Notably, PCR is probably a promising method but remains to be further improved. All of the methods mentioned above play important roles in various stages of syphilis. This review aims to provide a summary of the performance characteristics of detection methods for syphilis.

## Introduction

Syphilis is a multi-stage disease caused by *Treponema pallidum* subsp. *pallidum* (*T. pallidum*). It is primarily transmitted sexually or vertically during pregnancy (Radolf et al., 2016; Peeling et al., 2017). In recent decades, syphilis has been prevalent worldwide, especially in Africa, Southeast Asia, Western Europe, Russia, and China, where it has caused severe public health problems. The incidence of syphilis among men who have sex with men (MSM) has been increasing substantially, in particular among those who are HIV-positive, despite efforts being made to eliminate the disease (Gong et al., 2014; Kidd et al., 2018; XS, 2019). To control the spread of syphilis effectively, an accurate and efficient diagnostic tool for syphilis is particularly important.

In the absence of treatment, the natural history of syphilis is divided into primary, secondary, latent, and tertiary stages. Primary syphilis is characterized by the appearance of chancres on the genitals or inguinal lymphadenopathy that contains a high bacterial burden; these are generally painless and resolve spontaneously. In clinical practice, patients seeking medical advice are frequently in the secondary stage, which is characterized by maculopapular rash on the shoulders, arm, chest, or back and a condyloma lata in the perianal area. As signs and symptoms subside, patients enter a latent phase that can last for many years. Some untreated infected patients develop tertiary syphilis, characterized by destructive visceral, cardiovascular, or neurological disorders as well as severe skin lesions. Often, symptoms of tertiary syphilis occur 10 to 20 years after the initial infection. With widespread use of antibiotics, tertiary syphilis may become less common (Peeling et al., 2017). The diagnosis relies on history, physical examination, and interpretation of laboratory tests. Although *T. pallidum* is difficult to grow in culture, there are many direct and indirect tests for the spirochete. In primary syphilis the diagnostic criteria are based on positive darkfield result or polymerase chain reaction (PCR) of material from chancres, or a combination of a clinical diagnosis and positive serologic tests. Secondary syphilis is diagnosed using positive darkfield examination and reactive treponemal or alternative non-treponemal tests. Regrettably, in some stages, the disease may be asymptomatic, generating difficulty in diagnosing very early syphilis, neurosyphilis, and tertiary syphilis (**Table 1**) (Tuddenham et al., 2020).

**Table 1:** The comments of diagnostic methods in different stages of syphilis.

Stage	Diagnostic methods	Comments
Primary syphilis	DFM PCR serology	DFM of *T. pallidum* in chancre specimens have high specificity, but negative result does not rule out infection. In the window-period, serology may be negative in many patients. TTs are recommended in early primary syphilis. PCR-based tests have high dependability. Patients symptoms and medical history should be considered.
Secondary syphilis	DFM PCR IHC serology	*T. pallidum* in skin and mucosal lesions can be detected by DFM. PCR-based test and IHC may be useful for the diagnosis of secondary syphilis. Serology are intrinsically sensitive. Patients symptoms and medical history should be considered.
Latent syphilis	serology	NTTs have high sensitivity in early latent syphilis, but the sensitivity gradually decreased over time. TTs require confirmation in the presence of a negative result of NTTs.
Tertiary syphilis	serology	TTs should always be considered because some of the patient samples may present negative in NTTs. Patients symptoms and medical history should be considered.

To minimize the current diagnostic limitations, various detection methods for *T. pallidum* have been developed (Çakmak et al., 2019). The available tools include culture, morphological observation, immunohistochemistry (IHC), seroassay, and the nucleic acid amplification technique (NAAT). Culture is classically defined as the reference method for the detection of pathogenic agents; it can be subdivided into *in vivo* and *in vitro*. The rabbit infectivity test (RIT) is a typical *in vivo* culture technique that presents symptoms similar to those of humans. Unlike *in vivo* culture, *in vitro* culture for fastidious microorganisms is difficult. Fortunately, a recent study shown that *T. pallidum* was detectable in a cell culture system using a modified medium in a microaerophilic environment. The spirochetes are co-cocultured with Sf1Ep cells that are better at supporting the growth of *T. pallidum* than are other cells (Edmondson et al., 2018). Nevertheless, the cell culture system method is in a nascent stage; therefore the rabbit model remains the main method detection of *T. pallidum* isolated from suspected samples. The culture method should be accompanied by dark-field microscopy (DFM) and serologic tests to identify the spirochete. The morphological method, which is of high specificity in primary syphilis, relies on the particular wavy structure of spirochetes (Wolgemuth, 2015). These methods were developed using a multistage process. Coles et al. showed that darkfield illumination can be used to detect spirochetes (Coles, 1909). The direct fluorescent antibody staining for *T. pallidum* (DFA-TP) was developed to identify the presence of *T. pallidum* specimens in lesions or tissues (Ito et al., 1992). Non-treponemal tests (NTT) for syphilis have also been used. The first treponemal tests (TT) were made in 1949 by Nelson and Mayer (Nelson and Mayer, 1949). Currently, diagnosis remains primarily dependent on serologic tests, which have shown high sensitivity and specificity during secondary and early latent stages. With the emergence of *T. pallidum* whole nucleotide sequence (Weinstock et al., 1998), many investigators set out to introduce molecular biological techniques with some success. PCR is a promising technology for confirming a diagnosis of syphilis, especially for congenital syphilis, neurosyphilis, and primary syphilis. Each method has its own merits and defects. Descriptions of the various methods are detailed below.

## Morphology

*T. pallidum* is a delicate spiral organism with a hard, uniform, tight, and deep helix. The characteristic motion of *T. pallidum* is a forward and backward movement around the longitudinal axis. Due to the specific spiral-like shape of *T. pallidum*, morphological tests have been used to screen for primary syphilis in patients with chancre only using optical microscopy without other special instruments (Wolgemuth, 2015). The sensitivity vary from 71 to 100% depending on the sample (Forrestel et al., 2020). Morphological tests, although they require well-trained laboratory personnel and the identification of the results can be affected by subjective interpretation, have been routinely adopted by many laboratories to detect chancre samples from patients because of its simplicity, rapidity, and low price. Furthermore, DFM is inappropriate for detection of oral or rectal swabs because of the possibility that symbiotic treponema with high similarity may exist in these samples (Peeling et al., 2017). Nevertheless, a negative result does not exclude the diagnosis of syphilis because the chancres may resolve spontaneously and few organisms may be observed.

On the basis of DFM, silver-staining was developed for microscopic observation of bacteria. Silver-staining has been evaluated for the detection of *T. pallidum* in formalin-fixed paraffin-embedded (FFEP) tissue biopsies, principally from primary and secondary lesion biopsies (CG et al., 2004). Silver-staining eliminates the interference of mucous filaments and fibers; however, staining of melanin and reticulin fibers can mimic the appearance of spirochetes. Due to the challenges associated with stain interpretation and limited sensitivity, silver-staining is not routinely used in the diagnosis of syphilis.

The unsatisfactory specificity of DFM and silver-staining led to the development of DFA that detects pathogenic treponema using an antigen-antibody reaction (Ito et al., 1992). It is an immunofluorescence enzyme-based microscopic method that is suitable for lesion smears, concentrated fluids, and tissue brushings (Hook et al., 1985). The specificity of the method depends on the type of antibody used. The H9-1 monoclonal antibody is specific for *T. pallidum*, and does not react with other commensal spirochetes (Hook et al., 1985). For these reasons, DFA is used for samples from oral, rectal, and intestinal lesions with limited risk of false-positive results (Tsang et al., 2015). DFA is intrinsically specific and as sensitive as DFM for the diagnosis of early syphilis (Theel et al., 2020). However, a major limitation of this test is the availability of reliable specific anti-*T. pallidum* antibodies. Although such antibodies may be available commercially, none is currently ideal for clinical laboratories (Morshed, 2014). Another substantial obstacle is the unavailability of the reagents for DFA in remote areas such as underdeveloped countries. These studies were carried out decades ago, and no articles have been published recently to evaluate DFA, exacerbating the limited availability of reagents and the practicability of this method in current clinical practice.

## Immunohistochemistry

IHC directed against *T. pallidum* using fluorescent anti*-T. pallidum* antibodies has improved both the sensitivity and specificity of detection. Several immunohistochemistry techniques have been evaluated in FFPE tissue biopsies, especially the lesion biopsies from secondary syphilis. The avidin-biotin peroxidase complex (ABC) technique is the most frequently form of this assay (Theel et al., 2020). In short, this method involves exposing epitope of T. pallidum in tissue biopsies by heat induction, followed by incubating with rabbit anti*-T. pallidum* immunoglobulin G (IgG) antibodies. Subsequently, biotinylated antibodies to rabbit IgG are added, and finally are incubated with peroxidase-conjugated avidin-biotin complex and observation of *T. pallidum*. The sensitivity of IHC method range from 49 to 92% for the diagnosis of secondary syphilis, with excellent specificity (Müller et al., 2011). IHC is more sensitive and specific than silver-staining for detecting *T. pallidum* in biopsies from patients with secondary. When serologic assays failed to detect *T. pallidum* antibodies, IHC is a useful complementary diagnostic tool. However, *T. pallidum* can cross-react with other spirochetes, including *Borrelia burgdorferi* and the intestinal spirochetes (Graham et al., 2018). **Table 2** is a summary of the direct detection tests used for syphilis diagnosis.

**Table 2:** Direct detection tests for *T. pallidum*.

Method	Specimens	Advantages	Limitations	Reference no.
DFM	Chancres or cutaneous lesions	Direct detection of the specific spiral-like shape of *T. pallidum*	Requires specialized laboratory staff Should not be used to detect oral or rectal specimens Less sensitive Subjective	(Forrestel et al., 2020) (Peeling et al., 2017)
Silver-staining	Chancres or cutaneous lesions	More sensitive than DFM Eliminates the interference of mucous filaments and fibers	Requires specialized laboratory staff Less sensitive Subjective	(CG et al., 2004)
DFA	Chancres, cutaneous lesions, oral and rectal lesions	Intrinsically specific Can be used for oral, rectal, or intestinal lesions	Requires specialized laboratory staff Requires monoclonal-antibody reagent Subjective	(Hook et al., 1985; Larsen et al., 1995; Tsang et al., 2015)
IHC	Lesion biopsies (FFPE)	Excellent specificity in the diagnosis of secondary syphilis	Requires specialized equipment and stains Cross-react with other spirochetes Subjective	(Müller et al., 2011; Theel et al., 2020)

## Rabbit Infectivity Test

Several animals, including hamsters, chimpanzees, and rabbits, have been attempted to maintain treponema and determine infectivity. Eventually, the rabbit became the most practicable animal model because visible changes can be perceived with the naked eye and serologic tests for syphilis also become reactive. Classically, the reference method for direct detection of *T. pallidum* has been the RIT. First, suspected samples such as cerebrospinal fluid (CSF) or tissue fluid are injected into the testes of seronegative New Zealand white male mature rabbits. At 1 week after inoculation, the testes are inspected and the rabbit is bled for serologic testing using rapid plasma reagin (RPR) and *T. pallidum* particle aggutination (TPPA) every 2 days for the first month and every week for the next 2 months. If the animals become seropositive, the rabbit model is considered positive and motile spirochetes are observed in the testicular biopsies under DFM. Samples from the testes or lymph nodes are transferred to a new rabbit (Tong et al., 2017). Despite the high sensitivity of RIT, this method requires strict technical requirements in every step as well as the work of professional technicians. There are also ethical problems surrounding damage to rabbit testes. In addition, the observation of the changes in disease progression is time-consuming (Tsang et al., 2015). In this regard, RIT has been discontinued from most laboratories. Tong et al. showed that RIT is no longer highly sensitive for detecting *T. pallidum* in clinical samples as it had been previously, and is no longer considered a reference method for measuring the sensitivity of other methods in current antibiotic era (Tong et al., 2017). Nevertheless, it is undeniable that RIT is useful for recovering *T. pallidum* from infected tissue and maintaining viable spirochetes for research settings.

## Cell Culture System

In recent years, many efforts have been made to culture *T. pallidum in vitro*. If this can be achieved, it would not only facilitate the study of its physiology, gene, and pathogenesis, but it would also accelerate immunologic studies and vaccine development. Culture *in vitro* has been a persistent challenge. Fortunately, the culture technology based on modified medium and rabbit epithelial cell co-incubation system has been applied successfully to the Nichols strain of *T. pallidum*. Two additional newly *T. pallidum* isolates (UW231B and UW249B) were obtained as a result of substantial efforts (Edmondson et al., 2018). Cell culture systems were initiated using frozen preparations of *T. pallidum* extracted in advance from infected rabbit testes. *T. pallidum* and Sf1Ep cells were co-incubated in TPCM-2 in a microaerobic environment at 34°C, then the spirochetes were isolated from Sf1Ep cells using trypsin and EDTA. The suspension was observed under DFM and transferred to fresh subculture. Finally, rabbits were injected intradermally with bacterial suspension at marked sites along the back, and changes of cutaneous lesion were observed at intervals. The entire procedure requires rigorous attention to detail regarding the selection of tissue culture cells and the construction of a microaerobic environment. This may hamper its widespread use by laboratories. Consequently, the development of an axenic system that supports the long-term culture of *T. pallidum in vitro* remains the ultimate aim, and further simplification would be helpful for the investigation of these bacteria.

## Seroassays for Syphilis

Morphology tests based on the specific spiral-like shape of *T. pallidum* are helpful in the diagnosis of early syphilis; however, chancre specimens that contain several agents are often difficult to obtain because the lesion sites may resolve spontaneously. Currently, diagnosis remains primarily dependent on serologic evaluation for antibodies to *T. pallidum* using both NTT and TT (Larsen et al., 1995; Radolf et al., 2016; Peeling et al., 2017).

### Non-Treponemal Tests

NTTs, including the rapid plasma reagin test, toluidine red unheated serum test (TRUST), and the Venereal Disease Research Laboratory (VDRL) test, measure levels of anti-lipid immunoglobin M or G antibodies produced in response to lipoidal material released from damaged host cells or *T. pallidum* cardiolipin (Gao et al., 2018). Based on previous reports, the sensitivity of serum NTTs is 62–78% for the diagnosis of primary syphilis, 97–100% for secondary syphilis, and 82–100% for early latent syphilis. The sensitivity of serum NTTs for tertiary syphilis is unsatisfactory, ranging from 47% to 64% (Tuddenham et al., 2020). Despite the fact that there is relatively low sensitivity in the early stage due to the window period, or a prozone effect can occur in high-titer sera as an inappropriate proportion of antigen and antibody, NTTs have been routinely used because they are effective, simple to operate, inexpensive, and rapid (Morshed and Singh, 2015). Nevertheless, false positive results can occur in the context of pregnancy, autoimmune diseases, infections, and others (Gao et al., 2018).

Among the NTTs, the RPR and VDRL have an additional diagnostic value clinically. The RPR can be used for serum and plasma samples from patients at various stages, and can be performed in many laboratory settings. The VDRL is now primarily used for testing CSF from patients with neurosyphilis. The test has high specificity; however, the sensitivity is unsatisfactory (Marra et al., 2012). It is noteworthy that the criterion of the decline in the RPR titers to assess syphilis or neurosyphilis treatment has been recommended by a few guidelines (Janier et al., 2014; Xiao et al., 2017a). Nevertheless, a study showed that virulent *T. pallidum* remained viable in a patient with a negative serum RPR test after treatment (Lin et al., 2016). Recently, Lin et al. showed that the changes of RPR titers displayed a similar trend in the benzathine penicillin G (BPG) treatment group and the natural courses of syphilis, and therefore, RPR titers as the indicator of syphilis treatment efficacy become suspicious (Lin et al., 2020). Nevertheless, the RPR remains the classic index of clinical efficacy.

### Treponemal Tests

TTs, including the fluorescent treponemal antibody absorbed (FTA-ABS) test, *T. pallidum* particle agglutination, *T. pallidum* hemagglutination (TPHA) assays, enzyme immunoassay (EIA), and chemiluminescence immunoassay (CIA), detect antibodies against *T. pallidum* proteins directly and are supposed to possess high sensitivity and specificity. All immunoassays are almost 100% sensitive in secondary syphilis, 95.2–100% sensitive in early latent syphilis, and 86.8–98.5% sensitive in late latent syphilis (Park et al., 2019). TTs may be useful to make up for the deficiencies of NTT in early syphilis, because TT results might become positive once the primary chancre appears. Unfortunately, none of the TTs is helpful in evaluating treatment efficacy and they cannot distinguish active stage from a previously treated infection because treponemal antibodies in the patients with syphilis might persist throughout life.

The FTA-ABS is an indirect immunofluorescent staining assay. Nichols strain antigens are exposed to the suspected serum after the serum has been mixed with a sorbent (an extract from a nonpathogenic Reiter treponema). FITC-labeled anti-human immunoglobulin is added and combines with the patient’s antibodies. In a positive result, the presence of antibodies to *T. pallidum* are visible by fluorescence microscopy (Larsen et al., 1995). The FTA-ABS test is highly sensitive and specific; however, it may produce variable results due to variations in equipment, reagents, and interpretation. TPPA and TPHA are indirect agglutination tests in which surface antigens extracted from the whole *T. pallidum* are coated onto red cells or gelatin particles and react with the serum. The sensitivity of TPPA and TPHA ranges from 82 to 100% and the specificity is 99% (Peeling et al., 2017). The TPPA test, one TT that is widely used in laboratories, is cheaper and simpler than the FTA-ABS (Morshed and Singh, 2015). With treponemal assays (e.g., EIA and CIA) gradually becoming available, these assays have allowed clinical laboratories to meet the increasing demands for syphilis screening, enhancing efficiency, and providing accurate results. Several automated commercially available treponemal CIAs including the Liaison CIA (Diasorin, Stillwater, MN, USA) (Knight et al., 2007) and the Architect syphilis CIA (Abbott, Wiesbaden, Germany) (Young et al., 2009), and treponemal EIAs such as Captia syphilis G, Captia syphilis M (Trinity Biotech, Ireland), and Spiro Tek syphilis test (Organon Teknika, USA) (Ratnam, 2005), have been used in clinical laboratories (Qiu et al., 2015). These commercial products demonstrated excellent sensitivity and specificity when evaluated as confirmatory test and as screening tests for syphilis in various patient populations.

### Diagnostic Algorithms

**Figure 1:**
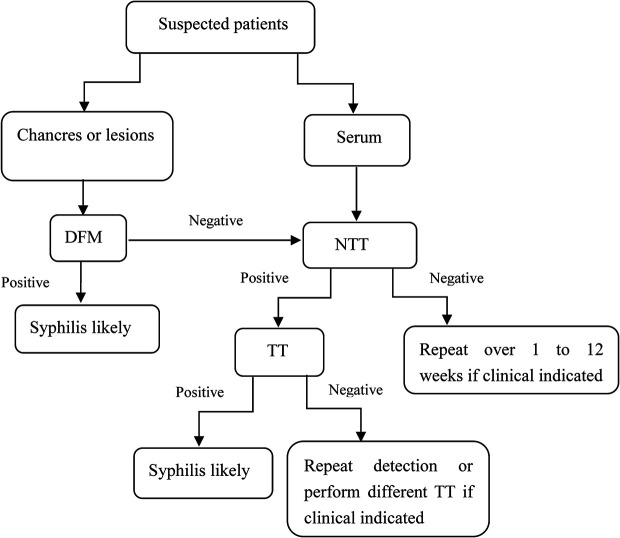
Testing algorithms for suspected patients.

**Figure 2:**
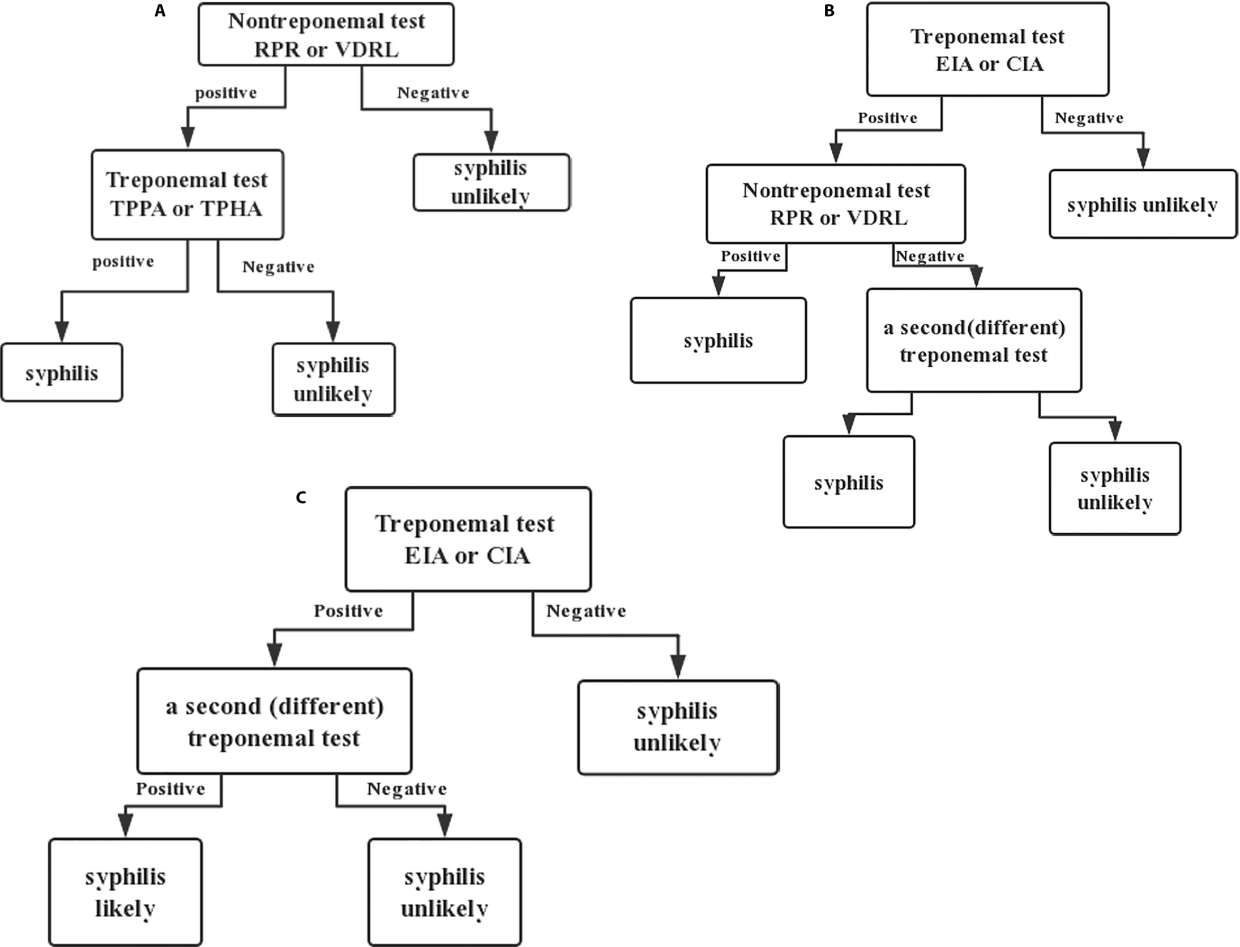
Syphilis testing algorithms. **(A)** Traditional, **(B)** Reverse, **(C)** ECDC.

Currently, serologic tests are the predominant methods for diagnosing syphilis in the laboratory (**Figure 1**). There are two commonly used algorithms used for the serological diagnosis of syphilis: the traditional algorithm (**Figure 2A**) and the reverse sequence serology algorithm (**Figure 2B**). Traditionally, an NTT, such as RPR or VDRL, is followed by a specific TT. However, many laboratories in America and Europe have changed their diagnostic approaches and adopted new procedures called reverse sequence serology algorithms (Morshed and Singh, 2015). The reverse algorithm uses one TT (such as CIA or EIA) with recombinant antigens to screen suspected samples, then positive samples are followed by a quantitative RPR or TPPA. If the results of these two methods are inconsistent, the specimen is retested using another treponemal test (Seña et al., 2010). Recently, European Centre for Disease Prevention and Control (ECDC) re-modified the reverse algorithm. The new algorithm called for using one TT first; positive results are then confirmed using another different TT (**Figure 2C**). Tong et al. found the traditional algorithm had a sensitivity of 75.81%, while the reverse algorithm and ECDC algorithm had higher diagnostic efficacy than did the traditional algorithm (Tong et al., 2014). Meeting all these testing criteria is not simple, and each of the previously described testing algorithms has its advantages and limitations. The traditional algorithm is affected by the subjective judgment of operator for NTTs. Second, the prozone effect can lead to a false-negative NTT result. Finally, the traditional algorithm is not always followed by TT in many laboratories, especially in resource-limited institutions. Therefore, some patients with early syphilis may be missed. By contrast, the reverse screening algorithm is helpful in high-volume screening of specimens, and false-negative results caused by prozone effect do not occur. Though it has its own limitations. Importantly, initial setup costs and ongoing operational costs may be higher than those of the traditional algorithm (Ortiz et al., 2020). Furthermore, a false-positive result may lead to potential overtreatment. In addition, in high-risk populations, algorithm that screen using TT can give rise to high rates of positive results, causing increased clinician workload (Janier et al., 2014). None of the seroassays can diagnose syphilis alone. Therefore, all screening results must be combined with clinical presentation. Considering the pros and cons of each algorithm, using one NTT or TT as the first screening tests should be based on factors including local prevalence, the clinical laboratory workload, experimental equipment, and related budget.

Although seroassays are associated with high sensitivity during secondary and later stages of syphilis, the sensitivity of these tests to detect primary syphilis is disappointingly low. Other limitations include lifelong positive results using TTs, the probability of serafast status, and incomprehensible results. Hence, they are not sufficient to meet clinical needs. It remains unclear whether there is an ideal serological marker for the evaluation of therapeutic effect. Recently, recombinant antigens based on *T. pallidum* outer membrane proteins (OMPs) were developed and were preliminarily applied to syphilis serological diagnosis. Tp0971 is one such OMP that is expected to replace RPR as a marker for the detection and evaluation of therapeutic effect. Liu et al. demonstrated that Tp0971, an infection phase-dependent antigen, is a promising diagnostic antigen for syphilis that may be used to monitor treatment (Liu et al., 2019). Assuming that this finding is confirmed, it will bring significant changes to the monitoring of syphilis treatment and will compensate for the deficiencies of current serologic tests.

## Nucleic Acid Amplification Technique

NAAT is a popular way to diagnose infectious diseases, especially for diseases caused by agents that are difficult to be cultured, such as *Borrelia burgdorferi* (the causative agent of Lyme disease) (Shah et al., 2018) and *Mycobacterium tuberculosis* (Khosravi et al., 2017). Because *T. pallidum* is so difficult to grow in culture, it is of great value to apply NAAT to the detection of *T. pallidum* (Morshed et al., 2007). Some studies showed that five types of NAAT, including routine PCR (Buffet et al., 2007; Shields et al., 2012; Gayet-Ageron et al., 2013; Gayet-Ageron et al., 2015b), nested PCR (nPCR) (Grange et al., 2012; Gayet-Ageron et al., 2015a; Vanhaecke et al., 2016; Wang et al., 2018; Guan et al., 2019), real-time PCR (qPCR) (Heymans et al., 2010; Tipple et al., 2011; Dubourg et al., 2015), reverse transcription PCR (RT-PCR) (Centurion-Lara et al., 1997), and loop-mediated isothermal amplification (LAMP) assay (Xiao et al., 2017b) could be used to diagnose syphilis. The target genes used for NAATs include *polA*, t*pp47*, *bmp*, *16S rRNA gene, tmpC*, and *tmpA.* Notably, *polA*, *tpp47*, and *bmp* are main target genes (**Table 3**) (Centurion-Lara et al., 1997; Gayet-Ageron et al., 2013; Gayet-Ageron et al., 2015a; Xiao et al., 2017b).

**Table 3:** NAATs for *T. pallidum.*

Type of NAAT	Stage	Specimen	Target gene	Sensitivity (%)	Specificity (%)	Reference no.
Routine PCR	Primary	Ulcer	Tpp47	87.0	93.1	(Gayet-Ageron et al., 2012b)
		Lesion	Tpp47	89.1	99.1	(Shields et al., 2012)
		Whole blood		36.1	95.7	(Gayet-Ageron et al., 2013)
	Secondary	Lesion	Tpp47	50	100	(Shields et al., 2012)
		Whole blood		54.2	93.2	(Gayet-Ageron et al., 2013)
	Neurosyphilis	CSF	Tpp47	75.8	86.8	(Castro et al., 2016)
			polA	69.7	92.3	
Nest PCR	Primary	Ulcer	Tpp47	79.8	95	(Grange et al., 2012)
			polA	71.4	93.7	
		Whole blood	Tpp47	53.6		
	Secondary	Whole blood	polA	62.9		(Wang et al., 2018)
	Latent	Whole blood		7.4		
	Neurosyphilis	CSF		42.5	97	(Vanhaecke et al., 2016)
Real-time PCR	Early infection	Ulcer	Tpp47	100	97.14	(Tipple et al., 2011)
		Blood	Tpp47	34.1	100	
	Secondary	Urine	polA	16		(Dubourg et al., 2015)
RT-PCR		CSF	16sRNA			(Centurion-Lara et al., 1997)
LAMP	Secondary	Peripheral blood	bmp	82.1	100	(Xiao et al., 2017b)

The samples used for NAAT are generally taken from lesion sites, including genital, anal, or oral ulcers, or surface rashes, tissue lesions, and mucosal erosion. The sensitivity and specificity of NAAT vary depending on the method, as well as the stage of syphilis. Some studies demonstrated that the sensitivity and specificity of routine PCR range from 78.4 to 89.1% and 93.1 to 100% in primary syphilis. It should be noted that the sensitivity of routine PCR is 89.1% in chancre specimens from primary syphilis (Gayet-Ageron et al., 2013; Gayet-Ageron et al., 2015b). In secondary syphilis, however, it has low sensitivity and specificity (Shields et al., 2012). Therefore, except for ulcer specimens of primary syphilis, the sensitivity of syphilis specimens in other stages is unsatisfactory. The US Centers for Disease Control and Prevention updated the definitions for the diagnosis of primary syphilis, in which PCR is regarded as a valuable diagnostic method, especially for use with chancre samples (Gayet-Ageron et al., 2015b). Nevertheless, a single study reported that the sensitivity of PCR targeting *tpp47* gene to detect frozen skin biopsies from patients with secondary syphilis was 72%, which was higher than that of other ordinary samples (Buffet et al., 2007). If possible, further studies should be focused on whether frozen skin biopsies have the ability to diagnose patients with secondary syphilis.

Because lesion specimens are difficult to obtain, researchers turned to fluid specimens such as blood, urine, and saliva. To increase the sensitivity of body fluid samples, nPCR was used to detect *T. pallidum* in whole blood samples from patients with syphilis. This is a relatively simple and adaptable method that involves two pairs of primers to amplify the target gene; nPCR significantly improves the specificity and sensitivity over those of routine PCR (Wills et al., 2018). It is reported that *tpp47* and *polA* are frequently used target genes for nPCR. One study shows that, when DFM is taken as a reference method, PCR targeting *tpp47* and *polA* gene had similar accuracy for the diagnosis of early syphilis (Gayet-Ageron et al., 2015a). The nPCR is also a sensitive test for *T. pallidum* DNA (Tp-DNA) in primary and secondary syphilis, whereas the sensitivity in latent syphilis is low. Researchers found that the sensitivity of nPCR ranged from 53.6 to 62.9% in blood (Wang et al., 2018), while the sensitivity of routine PCR ranged from 36.1 to 50.2%, suggesting that nPCR may be a worthy method for detecting Tp-DNA in the blood (Gayet-Ageron et al., 2013). Another investigation demonstrated that the positive rate of nPCR was 42% in CSF, whereas the positive rate of CSF VDRL was merely 30%. These findings suggest that nPCR has potential value in the diagnosis of neurosyphilis as well (Vanhaecke et al., 2016). Similarly, the sensitivity of nPCR for *tpp47* in whole blood from newborns with congenital syphilis was very high (Guan et al., 2019). In summary, nPCR is a sensitive method for detecting the syphilis in blood, and may be beneficial for diagnosing neurosyphilis.

To better understand the kinetics of *T. pallidum* infection, qPCR is performed. This technique quantifies the total amount in unknown samples using standard curves or performs relative quantification using the Ct value. It is difficult to calculate the DNA loads because the accuracy may rely on target gene, the type of sample, and extraction efficiency. Currently, qPCR targeting *polA* and *tpp47* has been used to detect syphilitic lesions, genital ulceration swabs, urine, and blood. Dubourg et al. found that the sensitivity of non-invasive (such as urine) specimens was lower than that of ulcer samples (Dubourg et al., 2015). The sensitivity of qPCR was almost 100% in ulcers, which is higher than that of blood samples (34.1%) (Tipple et al., 2011). The sensitivity and specificity of chancre samples from primary syphilis were 72.8 and 95.5%, respectively; however, the sensitivity was falsely low in secondary syphilis (Heymans et al., 2010). These findings suggest that qPCR might be useful for the detection of *T. pallidum* DNA in chancre swabs. qPCR, in spite of its efficiency and convenience, is not commonly used in the laboratory because it requires specific kits.

RT-PCR targeting *16S rRNA* is sensitive for detection in CSF (Centurion-Lara et al., 1997). The gene sequence of *16S rRNA*, including approximately 1,500 bps, usually contains conserved and variable regions. The frame of conserved regions is used to design universal primers to amplify genes in various groups, while the variable regions are useful for comparative taxonomy (Clarridge, 2004). RT-PCR distinguishes between living and dead *T. pallidum* by determining the degree of degradation of RNA after cell death. These studies were carried out decades ago, and no recent papers have been published to evaluate RT-PCR.

As mentioned above, to a large extent, the sensitivity of PCR depends on the bacterial loads; therefore it is imperative to increase the positive rate of NAAT. LAMP is a new NAAT that is rapid, simple, highly sensitive, and has no need for sophisticated devices. Papers have reported that this method can detect pathogens such as *Plasmodium* and *Schistosome* (Pöschl et al., 2010; Hamburger et al., 2013). However, LAMP is rarely used in the clinical laboratory as the availability needs further verification. Xiao et al. indicate that LAMP has better sensitivity and specificity in peripheral blood for the diagnosis of secondary syphilis, suggesting that LAMP has shown a bright prospect as a diagnostic test (Xiao et al., 2017b). Further studies are required to confirm this finding.

## Detection of *Treponema pallidum* Proteins

Mass spectrometry (MS) is an analytical technique in which chemical compounds are ionized into molecules and the ratio of mass to charge is measured (m/z) (Singhal et al., 2015). The method can be used for microbial identification, strain typing, and detection of antibiotic resistance. MS is fast and sensitive, and can detect polypeptides for the diagnosis of *leptospirosis* (Nally et al., 2015; Singhal et al., 2015). Considering little is known about the *T. pallidum* proteome, MS is needed to explore protein expression during infection. Recently investigators reported four unique *T. pallidum* proteins in four different urine pools from individuals infected with syphilis (Osbak et al., 2018) (**Table 4**). Unique proteins can be used to predict cellular localization and function, suggesting that this method might constitute a promising assay to detect *T. pallidum.* MS is infrequently used in the clinical laboratory because the data analysis is time-consuming and the equipment is costly. Nevertheless, this method may play an important role in the diagnosis of infectious diseases including syphilis in the future.

**Table 4:** Characteristics of four detected proteins.

Tp number	Protein name	Clinical stages
Tp0486	Borrelia-like antigen P83/100	Primary and secondary syphilis
Tp0742	GTPase Obg	Primary and secondary syphilis
Tp0804	ABC protein	Latent stage
Tp0369	Unknown protein	Primary and secondary syphilis

## Discussion

Rapid and accurate detection of syphilis is vital to ensure timely treatment and to control the transmission of the disease. Seroassays are the main diagnostic methods that have been widely adopted in clinical laboratories. Nevertheless, these methods have limitations in various stages, giving rise to the need for new diagnostic methods that are highly sensitive and specific.

Traditionally, the decline of RPR titers has been used to track recovery; however, a recent investigation case doubt on the notion that RPR titers predict treatment success because the clinical manifestations may heal spontaneously and RPR titers may also decline without therapy (Lin et al., 2020). Detection of infection phase-dependent antigens and better monitoring indexes will help alleviate this bottleneck. Another hurdle is finding the ideal diagnostic antigen for screening based on the combination of several factors, including detectable immunoreactivity, expression levels in various stages of syphilis, and decreased levels of immune response after the elimination of infection. The development of *T. pallidum* recombinant antigens remains a significant task, and the identification of immunodominant epitopes of infection phase-dependent antigens and synthesized polypeptides are other promising directions in the development recombinant antigens.

NAATs are promising diagnostic methods that reduce the turnaround times and are seldom affected by the subjectivity of the technologist. Gratifying achievements have been made in the development of NAAT. For example, a PCR-based method demonstrated satisfactory sensitivity in chancre samples and nPCR was helpful in blood (Shields et al., 2012; Wang et al., 2018). In conditional laboratories in America and Europe, PCR is used as a method for determining primary syphilis by detecting Tp-DNA from chancre specimens; however, the prerequisite is to ensure that the conditions of the laboratory must attain the corresponding standard. Unfortunately, PCR detection for secondary or latent syphilis is not satisfactory. We speculate that the reasons for the low sensitivity are as follows: 1) after entering the bloodstream, *T. pallidum* may attach to blood vessels, hide, or diffuse to organs to avoid the immune system; 2) the ideal target genes for PCR need to be further discovered; 3) substantial amounts of *T. pallidum* may be partially cleared by the host’s immune system after it enters the blood, and consequently the bacterial loads are reduced. Clinical sample preparation procedures and sample types also impact Tp-DNA loads. An appropriate centrifugation and red cell lysis pretreatment in whole blood improves the efficiency of DNA extraction. Identification of optimal target genes for *T. pallidum* and methods for enriching *T. pallidum* in blood are important tasks for future studies.

At present, the diagnosis for neurosyphilis remains a major clinical challenge. The laboratory diagnostic methods for neurosyphilis depend on CSF RPR test, CSF TPPA/FTA-ABS, CSF VLDL, CSF white blood cell counts, and CSF protein concentrations (**Table 5**). VLDL, performed on CSF, is generally considered specific for neurosyphilis, but it has limitations in terms of sensitivity and specificity. As a result, additional CSF testing and syphilis prevalence are required to diagnose neurosyphilis (Park et al., 2020). Many new potential biomarkers are warranted for discriminating neurosyphilis. Some cases illustrated that levels of CSF IL-10 and CXCL-13 in patients with neurosyphilis elevated significantly (Li et al., 2020; Gudowska-Sawczuk and Mroczko, 2020). Based on these findings, the production of cytokines and chemokine might be helpful for the diagnosis of neurosyphilis and to track the progression of the disease. Other biomarkers that can assist the diagnosis of neurosyphilis include microRNAs, which are characterized by their low immunogenicity and ability to cross the blood-brain barrier (Chen et al., 2019). Nevertheless, the expression of cytokines and chemokine as well as microRNAs remain to be studied and are not widely used in the clinic.

**Table 5:** Neurosyphilis diagnostic criteria.

Types	Diagnostic criteria	Reference no.
Asymptomatic patients	Positive serum TT results and Reactive CSF VDRL (or CSF TPPA ≧1:640 when the CSF VDRL is nonreactive) and Pleocytosis (CSF-WBC >5 cells/mL) or CSF-protein >45mg/dL in Non-HIV infected patients. Positive serum TT results and Reactive CSF VDRL (or CSF TPPA ≧1:640 when the CSF VDRL is nonreactive) AND CD4 cell count (CD4 <350 cells/mm^3^) and Pleocytosis (CSF-WBC >10 cells/mL) for HIV infected patients on antiretroviral therapy OR CD4 cell count (CD4 >350 cells/mm^3^) and Pleocytosis (CSF-WBC >20 cells/mL) in those not on antiretroviral therapy.	(Marra et al., 2017) (Guarner et al., 2015) (Tuddenham and Ghanem, 2018) (Marra et al., 2007) (Ghanem et al., 2009) (Gonzalez et al., 2019)
Symptomatic patients	Neurological symptoms and Positive serum TT results and Reactive CSF VDRL and Pleocytosis (CSF WBC >5 cells/mL) or CSF protein >45mg/dL.	

Despite the fact that various methods have been used to diagnose syphilis in the laboratory, the diagnosis of particular stages remains difficult. Primary syphilis is often missed because chancres are generally painless and resolve spontaneously, and serologic results in primary syphilis generally have poor sensitivity. Therefore, NAAT may play a major role in the diagnosis of primary syphilis. When untreated patients seek medical advice, they generally present with typical symptoms of secondary syphilis. The sensitivity of seroassay is desirable for secondary syphilis, but the performance of NAAT is unsatisfactory. Therefore, diagnosing secondary syphilis can be combined with seroassay, clinical features, and exposure history. About 30% patients samples with tertiary syphilis may present negative in NTTs, whereas those are almost always reactive. For this reason, TTs should always be considered in the serological diagnosis of tertiary stage. The sensitivity of seroassay and NAAT for tertiary syphilis is relatively low. Finally, tertiary syphilis is an uncommon stage in the current antibiotic era and samples are difficult to obtain to perform experimental studies.

With the development of technology, many new methods have shown potential for diagnosing *T. pallidum*; nevertheless, most of them are unsatisfactory. The exploration of *T. pallidum* recombinant proteins and the application of NAAT in the diagnosis of syphilis are priorities of future studies. We believe that the continuous development of technology and innovation will result in more rapid, accurate, and effective methods for the diagnosis of syphilis and as well for evaluation of therapeutic effects.

## Author Contributions

YL led the writing of the manuscript. YXiao and YXie developed the initial concept and framework from the manuscript and oversaw the drafting of the manuscript. All authors contributed to the article and approved the submitted version.

## Funding

This work was supported by the National Natural Science Foundation of China (81702046) and Natural Science Foundation of Hunan Province (2019JJ50535).

## Conflict of Interest

The authors declare that the research was conducted in the absence of any commercial or financial relationships that could be construed as a potential conflict of interest.
